# Multifocal thymic cysts with cholesterol granuloma

**DOI:** 10.1002/rcr2.361

**Published:** 2018-08-31

**Authors:** Shunichi Nagata, Misa Ishihara, Mitsugu Omasa, Takao Nakanishi, Hideki Motoyama

**Affiliations:** ^1^ Department of Thoracic Surgery Kobe City Nishi‐Kobe Medical Center Kobe Japan; ^2^ Department of Clinical Pathology Kobe City Nishi‐Kobe Medical Center Kobe Japan

**Keywords:** Cholesterol granuloma, mediastinum, thymic cyst, thymus

## Abstract

A 56‐year‐old female presented to our department with chest discomfort. Contrast‐enhanced computed tomography (CT) revealed a cyst and nodule in the anterior mediastinum; positron emission tomography–CT showed an increased uptake in the nodule. Total thymectomy was performed to obtain a definitive diagnosis and treatment. A pathological diagnosis of multifocal thymic cysts with cholesterol granuloma was made. Microscopic examination revealed different‐sized cysts scattered in the thymus. These cysts were filled with cholesterol clefts and manifested three different phase characters. The nodule comprised granuloma‐containing cholesterol clefts. We report a rare case of a patient whose histopathology presented a series of cholesterol granuloma formations.

## Introduction

The occurrence of cholesterol granuloma in the thymus is extremely rare. Although this condition is suggestive of an association with multilocular thymic cysts, the pathogenesis of cholesterol granuloma formation has not yet been proven 1. We report a rare case of a patient who histopathologically presented a series of thymic cholesterol granuloma formation.

## Case Report

**Figure 1:**
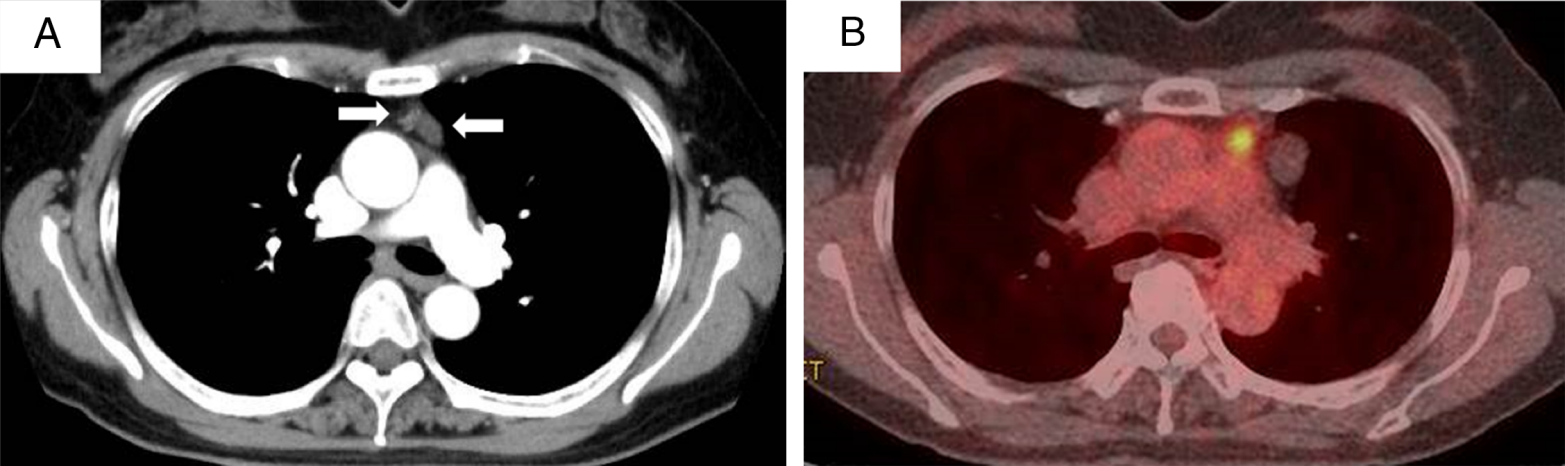
(A) Chest computed tomography (CT) showing a 35‐mm, well‐circumscribed cyst (arrow) and an adjacent 10‐mm nodule (arrow) in the anterior mediastinum. (B) FDG‐PET/CT showing increased uptake only in the 10‐mm nodule (arrow) in the anterior mediastinum (SUVmax 3.8).

A 56‐year‐old female presented to our department with chest discomfort. Contrast‐enhanced chest computed tomography (CT) revealed a 35‐mm well‐circumscribed cyst and an adjacent 10‐mm nodule in the anterior mediastinum (Fig. 1A). Positron emission tomography–CT demonstrated an increased uptake only in the small nodule (maximum standardized uptake value: 3.8; Fig. 1B). The patient took pitavastatin for hyperlipidaemia (triglyceride 271 mg/dL, LDL‐cholesterol 180 mg/dL) and had no evidence of cholesterol deposition in tissue or organ at the time. She had no history of trauma and was not on any anticoagulant drugs. The laboratory findings, including the tumour marker levels, were all within the normal range. Therefore, based on the diagnosis of cystic thymoma, total thymectomy was performed for a definitive diagnosis and treatment plan creation.

**Figure 2:**
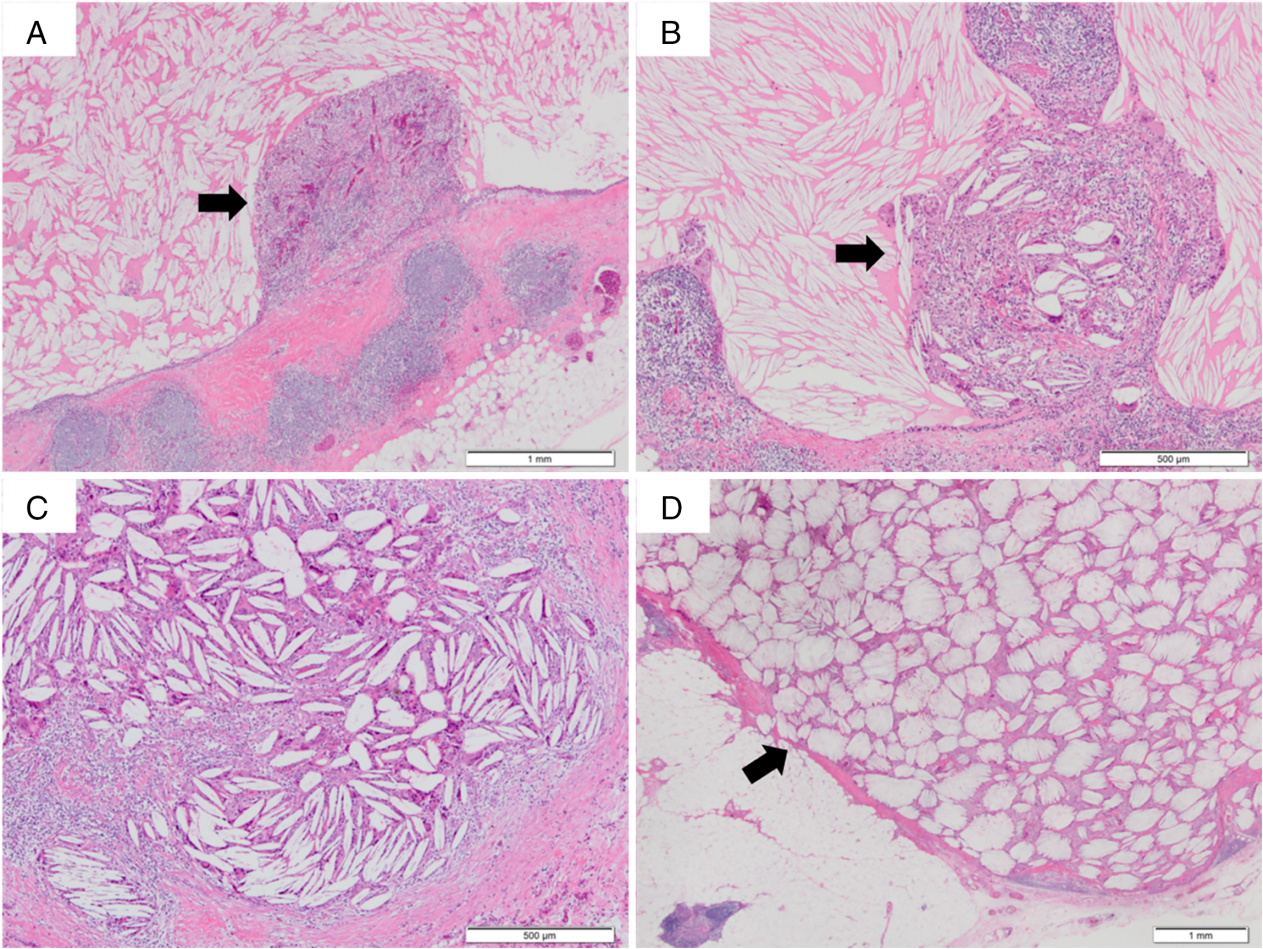
Pathological findings. (A) Inflammatory granulation (arrow) inside the ruptured cystic wall. (B) Granuloma involving cholesterol clefts (arrow) formed in the lining of the cystic wall. (C) Granuloma involving cholesterol clefts replacing lumen. (D) Cholesterol clefts involved in the granuloma arranged in an alveolar‐like growth pattern (arrow).

Macroscopically, a 20 × 20 × 10‐mm cyst containing yellowish‐brown jellied effusion and a 13 × 13 × 12‐mm, light‐brown, solid nodule were observed separately in the thymus. Microscopically, different‐sized cysts with foam cell infiltration were noted to be scattered in the thymus. Almost all cysts were filled with several cholesterol clefts and showed three different phase characters. Inflammatory granulation was noted inside the ruptured cystic wall (Fig. 2A). Granuloma involving cholesterol clefts was formed in the lining of the cystic wall (Fig. 2B), and this replaced the whole lumen (Fig. 2C). Conversely, the 10‐mm nodule, not bordering the thymic cysts, was composed of several granuloma‐containing cholesterol clefts without any cystic component. Cholesterol clefts in the granuloma were arranged in an alveolar‐like growth pattern (Fig. 2D), which was different from that in the lumen (Fig. 2C). Based on the above‐mentioned findings, the pathological diagnosis of multifocal thymic cysts with cholesterol granuloma was made. The patient was discharged without any post‐operative complication and was followed up for two years post‐operatively without any recurrence.

## Discussion

Thymic cysts, which account for <1% of mediastinal cystic lesions, are usually asymptomatic. They can be divided into congenital unilocular thymic cysts, which is the most common type, and multilocular thymic cysts, which result from an acquired inflammatory process. Although multilocular thymic cysts are sometimes associated with malignancy, unilocular thymic cysts are rare 1.

A foreign body‐type giant cell reaction incited by the deposition of cholesterol clefts leads to the formation of cholesterol granuloma 2, which is believed to be associated with bleeding, inflammation, or hyperlipidaemia 3. Cholesterol granuloma usually deposits in the breast and middle ear but very rarely in the thymus. Therefore, complete surgical resection is recommended because of its association with malignant diseases 4.

The inflammatory reaction around the ruptured cystic wall is a presumed pathogenesis of cholesterol granuloma 5; it has not been proven pathologically 1. In this case, the pathological findings indicated a process from the retention of cholesterol crystals within the multifocal thymic cysts to the cholesterol granuloma formation. The ruptured cystic wall provokes an inflammatory response that leads to the formation of inflammatory granulation tissues. The inflammatory granulation tissue spreading from the wall to the lumen involves cholesterol crystals derived from cyst fluid and gradually replaces the cystic lumen. The tissue finally detaches from the cystic wall and contains stromal components such as fibroblasts and collagen fibres. Our case demonstrated these characteristic findings through a histopathological study. This may be important in future consideration or diagnostics.

### Disclosure Statement

Appropriate written informed consent was obtained for the publication of this case report and accompanying images.

## References

[1] Suster S , and Rosai J . 1991 Multilocular thymic cyst: an acquired reactive process. Study of 18 cases. Am. J. Surg. Pathol. 15:388–398. 2006719

[2] Milton CM , and Bickerton RC . 1986 A review of maxillary sinus cholesterol granuloma. Br. J. Oral Maxillofac. Surg. 24:293–299. 2942184 10.1016/0266-4356(86)90096-3

[3] Francis GA , Johnson RL , Findlay JM , et al. 2005 Cerebral cholesterol granuloma in homozygous familial hypercholesterolemia. CMAJ 172:495–497. 15710941 10.1503/cmaj.1041152 PMC548411

[4] Furuhira C , Ohshima A , Shimada K , et al. 2004 A case of breast cholesterol granuloma accompanied by cancer. Breast Cancer 11:210–213. 15550870 10.1007/BF02968304

[5] Weissferdt A , Kalhor N , and Moran C . 2015 Primary thymic cholesteroloma: a clinicopathological correlation of four cases of an unusual benign lesion. Virchows Arch. 467:609–611. 26266777 10.1007/s00428-015-1822-8

